# A Rare Clinical Presentation of Darier's Disease

**DOI:** 10.1155/2013/419797

**Published:** 2013-03-20

**Authors:** Mybera Ferizi, Antigona Begolli-Gerqari, Bostjan Luzar, Fisnik Kurshumliu, Mergita Ferizi

**Affiliations:** ^1^Department of Dermatology, University Clinical Center, 10000 Pristina, Kosovo; ^2^Institute of Pathology, University of Ljubljana, 1000 Ljubljana, Slovenia; ^3^Institute of Pathology, University Clinical Center, 10000 Pristina, Kosovo; ^4^Medical Faculty, University of Pristina, 10000 Pristina, Kosovo

## Abstract

Darier's disease, also known as keratosis follicularis or dyskeratosis follicularis, is a rare disorder of keratinization. It is an autosomal dominant genodermatosis with high penetrance and variable expressivity. Its manifestation appears as hyperkeratotic papules, primarily affecting seborrheic areas on the head, neck, and thorax and less frequently on the oral mucosa. When oral manifestations are present, the palatal and alveolar mucosae are primarily affected. They are usually asymptomatic and are discovered in routine dental examination. Histologically, the lesions are presented as suprabasal clefts in the epithelium with acantholytic and dyskeratotic cells represented by “corps ronds and grains”. This paper reports a case of a 53-year-old woman that was admitted to our clinic with more than 10-year history of keratotic papules, presented on the hands and feet, nose, ears, genitalia, and whitish lesions on palatal mucosae.

## 1. Introduction

Darier's disease or keratosis follicularis is a rare autosomal dominant genodermatosis, which is characterized by greasy, crusted, keratotic, yellow brown warty papules and plaques particularly over seborrhoeic areas. Although this is a genetically transmitted disease according to a larger series, about 47% of patients had no clear family history, presumably because of incomplete penetrance [1]. The disease is caused by mutations in the ATP 2A gene, which encodes the sarcoendoplasmic reticulum Ca^2+^ ATPase [1]. This disease was first describe by Prince Marrow in 1886 and simultaneously by Darrier and White in 1889, independently. In 1917, the first case with oral manifestation was reported by Reenstierna [2].

The prevalence of this disorder in population is 1 : 100,000. The sex incidence is equal, although the males appear to be more severely affected than females [2–4]. The oral mucosa is affected in 50% of the cases [4], and lesions are usually asymptomatic and discovered during routine dental examination [5, 6]. Lesions are represented by multiple firm papules with normal, whitish, or reddish color, primarily affecting the palatal and alveolar mucosa. Histologically, the lesions are presented as suprabasal clefts in the epithelium, with acantholysis and dyskeratotic cells presented as “corps ronds” and “corps grains “Corps ronds” are larger structures usually presented in the granular layer and consist of irregular eccentric and sometimes pyknotic nuclei [7]. Precipitating factors include heat and humidity, mechanical trauma like friction, sunlight, and secondary bacterial infections [8]. Associated anomalies have been described in the literature, including mental retardation and psychosis [6].

## 2. Case Report

A 53-year-old woman was admitted to our clinic with more than 10-year history of keratotic papules. Papules were present on the back of her hands and feet. The nails of her hands and feet were also affected Figures 3 and 4. In the nose, scalp, and ears, were visible skin colored there papules, with a few mm in size, clearly localized by healthy skin (Figures 5(a), 5(b), and 5(c)).

Keratotic papules were present on lower back, including the perineum and vulvar region (Figures 6, 7(a), and 7(b)). Oral lesions were detected as white papules with a central depression (Figure 8).

Considering her signs and symptoms, clinicians arrived at differential diagnoses including Darier's disease and Hailey-Haily disease, Bazex syndrome, ichthyosiform dermatosis, Langerhans cell histiocytosis, or severe AD.

**Figure 1:**
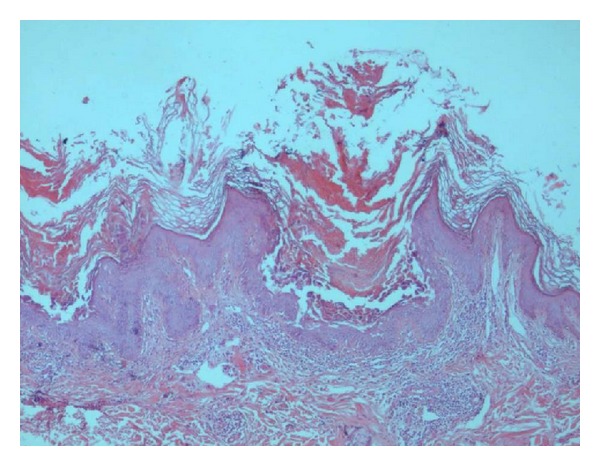
Epidermis with acanthosis premature keratosis and suprabasal acantholysis. A group of dyskeratotic cells (corps ronds and grains) is seen in the bottom of the suprabasal cleft (100x, H&E).

**Figure 2:**
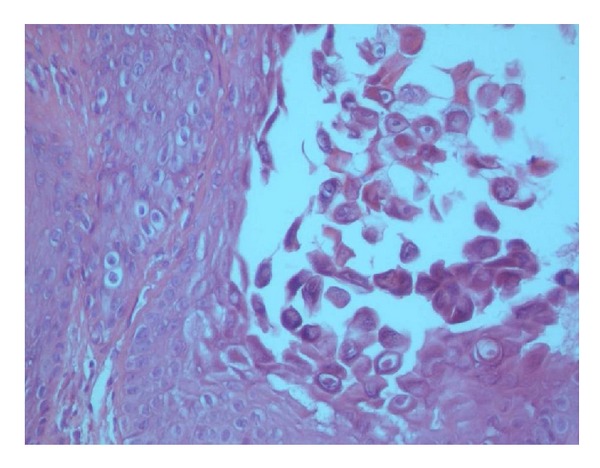
A group of dyskeratotic cells (corps ronds and grains) reflecting acantholysis and premature keratinization (200x, H&E).

**Figure 3:**
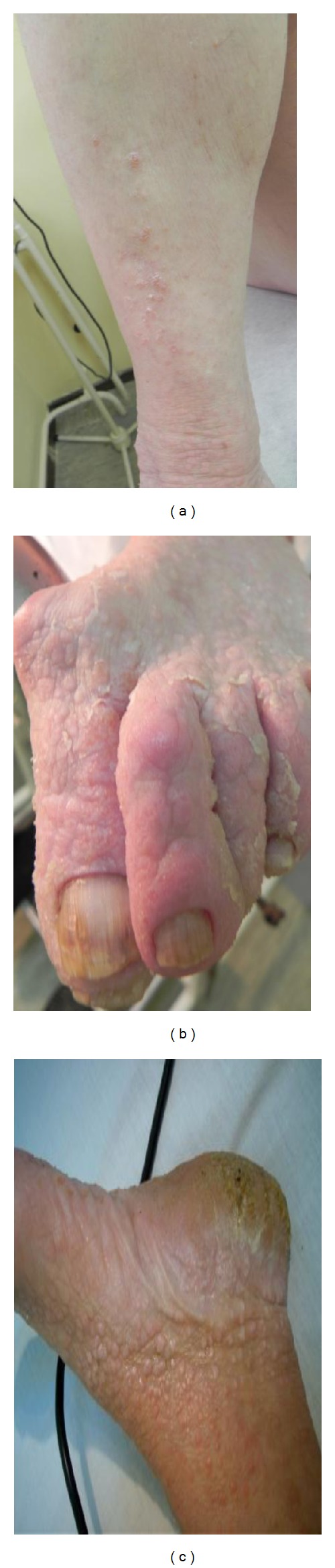


**Figure 4:**
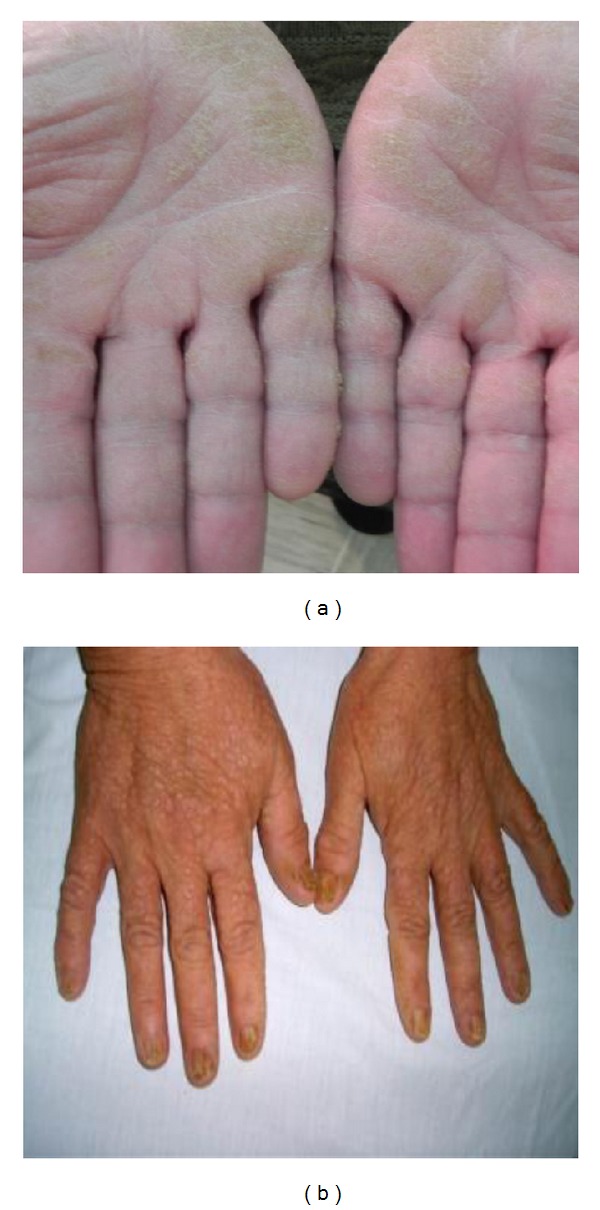
Similar papules were present on the back of her hands and feet. The nails of her hands and feet were also affected (Figures 3 and 4).

**Figure 5:**
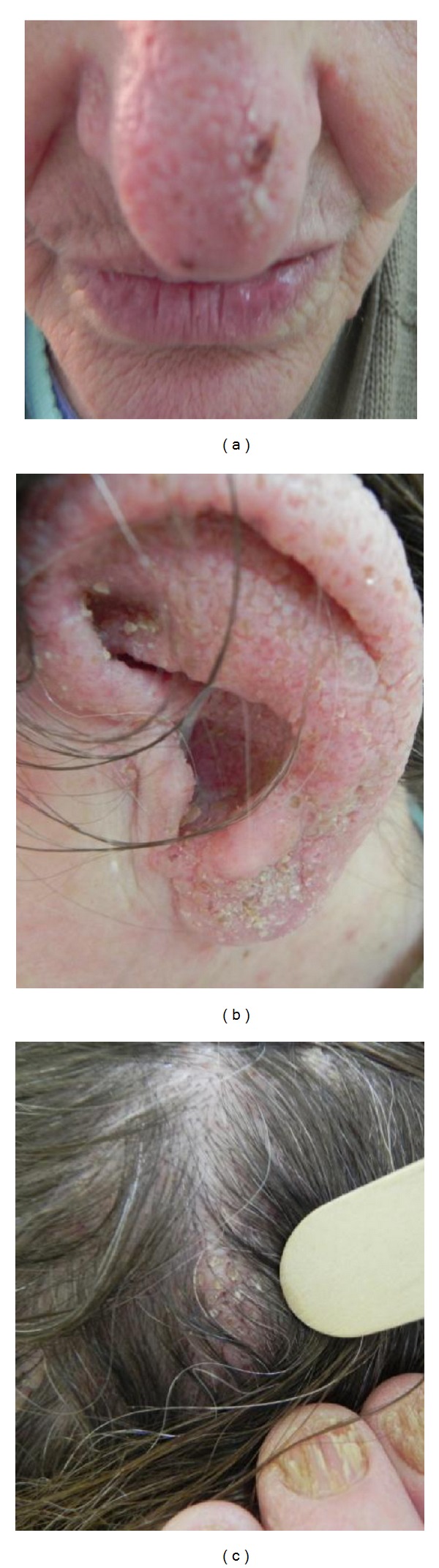
In the nose, ears, and scalp, are visible skin colored papules, with a few mm size, clearly localized by healthy skin (a, b, and c).

**Figure 6:**
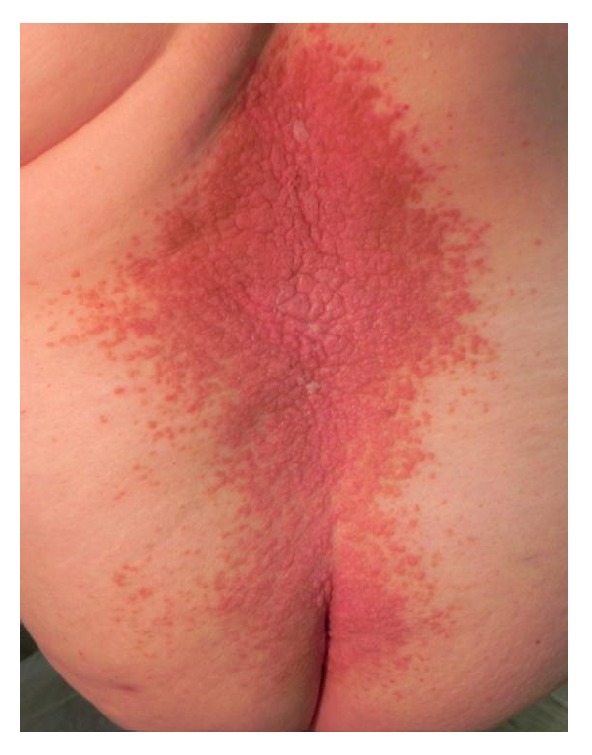
Keratotic papules are present on lower back.

**Figure 7:**
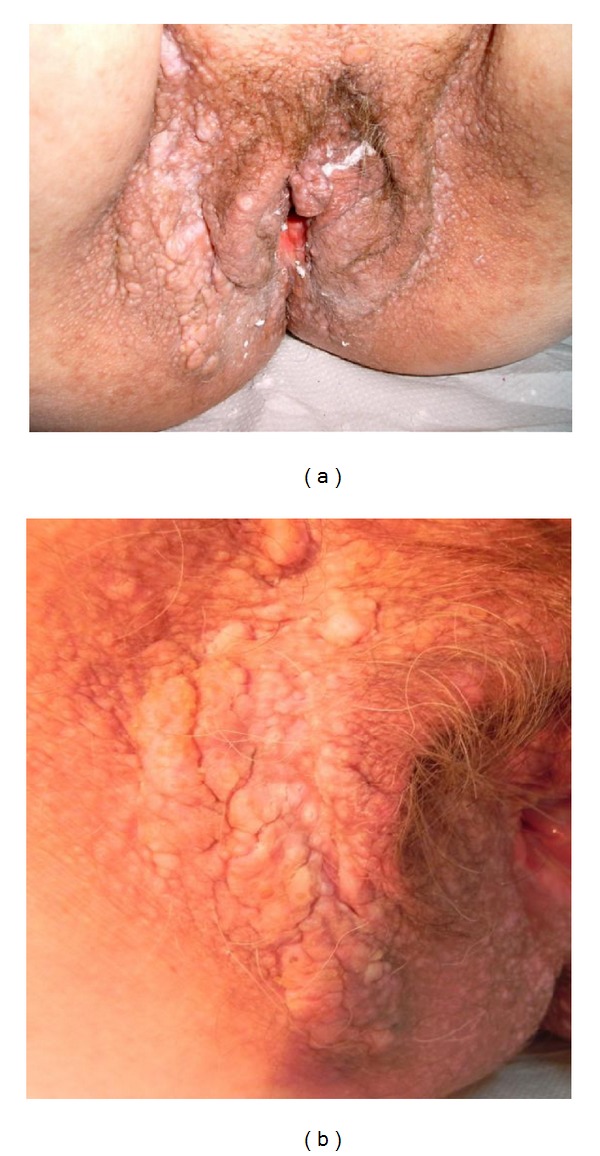
Papules also include the perineum and vulvae region.

**Figure 8:**
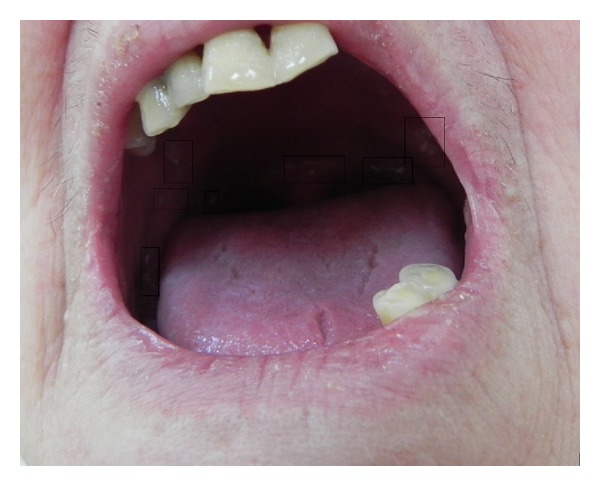
Oral lesions are detected as white papules with a central depression.

Histopathologically, the biopsy revealed the presence of epidermis with acanthosis premature keratosis and suprabasal acantholysis. A group of dyskeratotic cells (corps ronds and grains) is seen in the bottom of the suprabasal cleft. A group of dyskeratotic cells (corps ronds and grains) reflecting acantholysis and premature keratosis, was reported as keratosis follicularis by a multidisciplinary team (Figures 1 and 2).

Primary laboratory analysis of full blood found that liver enzymes and kidney function were normal, and the patient tested negative for syphilis and HIV infection. The vaginal and cervical smear showed presence of b-hemolytic streptococci. The PAP smear came back negative. Nail microscopic analysis was positive for fungi.

## 3. Discussion

Darier's disease is an autosomal dominant disease with high penetrance and variable expressivity. Although it is an inherited disease, 47% of the patients with Darier's disease do not have a family history [8]. Absence of family history could also be attributed to the fact that mild forms of the disease have not been recognized among the family members. Mutations in the ATP2A2 gene found on chromosome 12q that encodes for sarco/endoplasmic reticulum calcium ATPase pump (SERCA2) type 2 isoform are the cause of disease. Ca^2+^ ATPases are the key factors in the regulation of calcium in eukaryotic cells and are thus essential for correct functioning of the cell machinery [9]. Ca^2+^ ATPases transport Ca^2+^ from the cytosol back to the endoplasmic reticulum lumen hence mediate stability and adhesion of desmosomes. The mutations in this gene affect Ca^2+^ homeostasis and result in abnormality in desmosomal stability and adhesion [10].

Histologically, Darier's disease is characterized by acantholysis which forms suprabasal clefts and also formation of “corps rond and grains” superficially. Corps ronds are usually present in the granular cell layer and show central large round duskeratotic basophilic masses surrounded by a clear halolike zone. Darier's disease must be distinguished histologically from other acantholytic dyskeratoses, such as Haily-Haily disease (familial benign pemphigus) and Grover's disease (transient acantholytic dermatosis). In Haily-Haily disease, acantholysis is incomplete, causing the well-known “dilapidated brick wall” appearance of the lower epidermis [11]. The clinical characteristics of these diseases are different from those of Darier's disease.

Oral lesions are detected in approximately 15% of the patients, and they appear as white papules with a central depression [12]. Here, we report a rare clinical presentation with keratotic papules present on the hands and feet, nose, ears, genitalia, and whitish lesions on palatal mucosa. Similar papules were present on the back of her hands and feet. The nails of her hands and feet were also affected.

Darier's usually begins in the 4-5th decade of life, and our case also belongs to this category. The affected patient with oral lesions usually shows dry, crusted and itchy lesions on seborrheic areas, and similarly our case was also presented with crusted lips. Intraoral lesions are usually whitish and show variable consistency; similarly, this patient also had identical clinical features.

Although the present case is not a severe form of Darier's disease, most patients with severe form of Darier's disease should receive genetic counseling, including information of inherited condition and risk of transmission to offspring.

Biopsy is necessary to arrive at definitive diagnosis. Patients should be referred for dermatological examination and should be informed about the possible complications like bad odors, caries, and secondary infections. Psychiatric opinion should follow in more severe cases. Therefore, it is important to ensure multidisciplinary approach in the management of patients with Darier's disease.
